# Phrenic Nerve Stimulation Improves Physical Performance and Hypoxemia in Heart Failure Patients with Central Sleep Apnea

**DOI:** 10.3390/jcm10020202

**Published:** 2021-01-08

**Authors:** Max Potratz, Christian Sohns, Daniel Dumitrescu, Philipp Sommer, Henrik Fox

**Affiliations:** 1 Clinic for General and Interventional Cardiology/Angiology, Herz- und Diabeteszentrum NRW, Ruhr-Universität Bochum, 32545 Bad Oeynhausen, Germany; mpotratz@hdz-nrw.de (M.P.); ddumitrescu@hdz-nrw.de (D.D.); 2 Clinic for Electrophysiology, Herz- und Diabeteszentrum NRW, Ruhr-Universität Bochum, 32545 Bad Oeynhausen, Germany; psommer@hdz-nrw.de; 3 Heart Failure Department, Herz- und Diabeteszentrum NRW, Ruhr-Universität Bochum, 32545 Bad Oeynhausen, Germany; hfox@hdz-nrw.de; 4 Clinic for Thoracic and Cardiovascular Surgery, Herz- und Diabeteszentrum NRW, Ruhr-Universität Bochum, 32545 Bad Oeynhausen, Germany

**Keywords:** central sleep apnea, heart failure, phrenic nerve stimulation, six-minute walk test, hypoxia

## Abstract

Background: Central sleep apnea (CSA) is a common comorbidity in patients with heart failure (HF) and has been linked to increased morbidity and mortality risk. In addition, CSA is associated with impaired quality of life, reduced physical performance capacity, and hypoxemia. Phrenic nerve stimulation (PNS) is a novel approach to the treatment of CSA and has been shown to be safe and effective in this indication. However, there are currently no data on the effects of PNS on physical performance and hypoxia in CSA HF patients, both of which have been shown to be linked to mortality in HF. Methods: This prospective study enrolled patients with HF and CSA diagnosed using polysomnography. All were implanted with a PNS system (remedē^®^ system, Respicardia Inc., Minnetonka, MN, USA) for the treatment of CSA. Examinations included polysomnography (to determine hypoxemic burden), echocardiography and a standardized 6-min walk test prior to device implantation (baseline) and after 6 months of follow-up. Results: A total of 24 patients were enrolled (mean age 67.1 ± 11.2 years, 88% male). The 6-min walk distance was 369.5 ± 163.5 m at baseline and significantly improved during follow-up (to 410 ± 169.7 m; *p* = 0.035). Hypoxemic burden, determined based on time with oxygen saturation < 90% improved from 81 ± 55.8 min at baseline to 27.9 ± 42.8 min during PNS therapy (*p* < 0.01). Conclusion: In addition to safely and effectively treating CSA, PNS is also associated with improved physical performance capacity and reduced hypoxemic burden in patients with HF.

## 1. Introduction

Central sleep-disordered (CSA) breathing is a common comorbidity in patients with heart failure (HF) and studies have linked the presence of CSA to increased morbidity and mortality in these patients [1,2]. Other negative effects of CSA include increased arrythmia burden, impaired sleep quality, hypoxemia, and reduced functional physical performance capacity [1,3,4]. The adverse consequences of CSA, particularly in HF, can be attributed to episodes of hypoxia during apneas, which trigger oxidative stress, systemic inflammation, and endothelial dysfunction, further contributing to HF progression [5]. In addition, alternating phases of CSA hyperventilation result in sympathetic nerve activation, arousals and impaired sleep efficiency, and promote cardiac arrhythmia [5].

Another important impact of CSA in patients with HF is impairment of functional physical performance capacity [6]. To date, no treatment strategy has been shown to reverse or improve functional physical performance capacity in patients with CSA and HF. Data from two clinical trials have shown improvements in 6-min walk distance and cardiopulmonary exercise test performance during treatment of obstructive sleep apnea (OSA) in patients with HF [7,8]. With respect to CSA, only one randomized, controlled trial has hinted at a potential beneficial of CSA treatment on exercise capacity in HF patients with preserved ejection fraction [9]. The Cardiovascular Improvements With MV-ASV Therapy in Heart Failure (CAT-HF) trial showed some improvement in physical capacity, but only as part of a composite endpoint. Therefore, there is a need for more data in this area. Furthermore, hypoxemic burden has been shown to be the most robust predictor of mortality in HF patients with sleep apnea [10,11]. However, nothing is known about the effects of new emerging treatments such as phrenic nerve stimulation (PNS) on these surrogate endpoints for mortality in patients with HF.

PNS has been shown to be safe and effective for the treatment of CSA [12], with recent follow-up data showing enduring, safe, and reliable therapy effects for up to 36 months [13]. The system was designed to provide individualized, consistent, overnight CSA therapy, with a fully implantable device that delivers physiologic intrathoracic negative pressures rather than the more established masked-based positive airway pressure approaches, through transvenous unilateral phrenic nerve stimulation [13,14,15]. Although the PNS system is market approved and well described, nothing is known about the impact of PNS on functional physical performance capacity in patients with HF.

This prospective study investigated the effects of PNS on functional physical performance capacity and hypoxemic burden in patients with HF and CSA.

## 2. Methods

### 2.1. Study Design and Population

This prospective open-label study was conducted at our institution in Bad Oeynhausen, Germany over the period 2016 to 2020. The study protocol was approved by the local ethics committee (Ethikkommission der Ruhr-Universität Bochum, Sitz Ostwestfalen, AZ 2018-395) and all patients provided written informed consent prior to enrolment in the trial and before PNS implantation. Inclusion criteria were both HF with preserved or reduced ejection fraction when phrenic nerve stimulator was implanted for treatment of documented predominant central sleep apnea. Patients had to have optimal guideline-derived HF therapy for at least six month and all etiologies of heart failure were allowed for inclusion. Exclusion criteria were malignancies requiring treatment, end-stage renal disease, dialysis, or hemodynamically significant heart valve disease, as well as use of masked-based CSA therapies.

### 2.2. Patient Population

Patients had a confirmed HF diagnosis based on current guidelines [16], and had been receiving treatment with optimal guideline-based HF medication and cardiac devices [16] for at least 6 months. HF medication had to be unchanged before and after PNS implantation and throughout the study, with dose adjustments for hemodynamic tolerance only. CSA was defined and diagnosed using cardiorespiratory polysomnography in our sleep lab according to current American Academy of Sleep Medicine guidelines [17]. Patients were eligible for PNS implantation if the apnea-hypopnea index (AHI) was ≥20 /h, the proportion of central apneas was ≥50% and the central apnea index (CAI) was ≥30/h, and the proportion of obstructive apnea events was ≤20%. These criteria are in accordance with available large PNS trial, as recently reported [13].

### 2.3. Intervention and Follow-Up

Eligible patients were implanted with a PNS system (remedē^®^ system, Respicardia Inc, Minnetonka, MN, USA) for the treatment of CSA. All patients attended 6-monthly follow-up visits for assessment of treatment efficacy; these visits included echocardiography, polysomnography (PSG), and a 6-min walk test.

### 2.4. Cardiorespiratory Polysomnography

Sleep studies for CSA diagnosis were performed using in-hospital multichannel cardiorespiratory PSG (Somnomedics, Randersacker, Germany). Nasal air flow, chest and abdominal effort, pulse oximetry, and body position were recorded continuously.

### 2.5. Six-Minute Walk Test

Symptom-limited standardized six-minute walk test was consistently performed by all patients at baseline and during follow-up every six months. Patients walked the same standardized 50-m lane at their own pace for 6 min and the maximal distance walked was documented.

### 2.6. Primary and Secondary Outcome Parameters

Primary prespecified outcome was change in symptom-limited standardized six-minute walk test and hypoxemic burden in this patient population measure before PNS implantation and after six months of PNS. Secondary outcome parameters were changes in left ventricular ejection fraction (LVEF), left atrial diameter, BNP, CRP, creatinine, central respiratory events in total, central apnea index (/h), obstructive apnea index (/h), hypopnea index (/h), and apnea-hypopnea Index (/h). A reliable power calculation was not possible in advance as this is the first study on functional cardiovascular outcome parameters in the field.

### 2.7. Adverse Events

Occurrence of serious adverse events were determined at six-month post-baseline visits.

### 2.8. Statistical Analysis

Statistical analyses were performed using TIBCO Statistica 13.3 (3307 Hillview Avenue, Palo Alto, CA, USA). Data are expressed as percentages for categorical variables and as mean ± standard deviation (SD) or median with interquartile range (IQR) for continuous variables. Differences between normally distributed groups were compared using Shapiro–Wilk W test. Within-group differences were analyzed using paired *t*-test. For data with a non-normal distribution, Kruskal–Wallis was performed, and Wilcoxon signed-rank test was used for within-group comparisons. Statistical significance was defined as *p* < 0.05.

## 3. Results

A total of 24 patients were enrolled. The majority were male (92%), mean body mass index was high (34.6 ± 2.3 kg/m²) and 50% of patients were in New York Heart Association class III (Table 1). AHI at baseline was 38.1 ± 17.9 /h. The duration of the PNS implantation procedure was 137.6 ± 50.4 min. Full details of the procedure are summarized in Table 2.

### 3.1. Physical Performance

**Figure 1:**
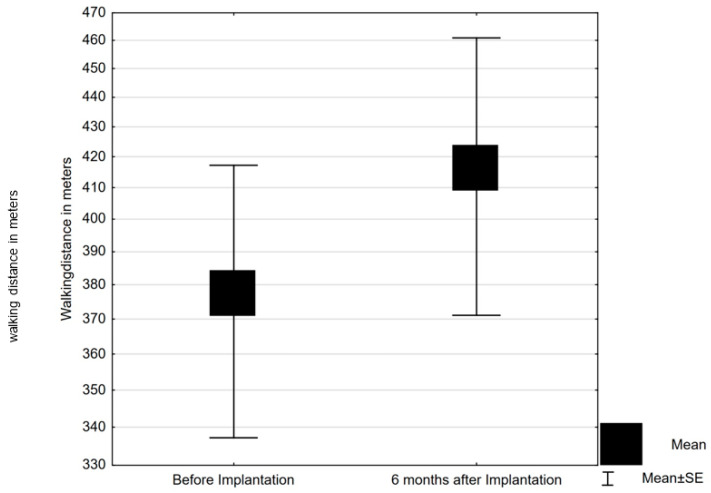
Standardized six-minute walking test distance at baseline and after 6 months of phrenic nerve stimulation (mean with standard error). SE, standard error.

Six-minute walk distance improved significantly from 369.5 ± 163.5 m at baseline to 410 ± 169.7 m at the 6-month follow-up under continuous PNS treatment (*p* = 0.035) (Table 3, Figure 1).

### 3.2. Respiratory Parameters and Hypoxemia

**Figure 2:**
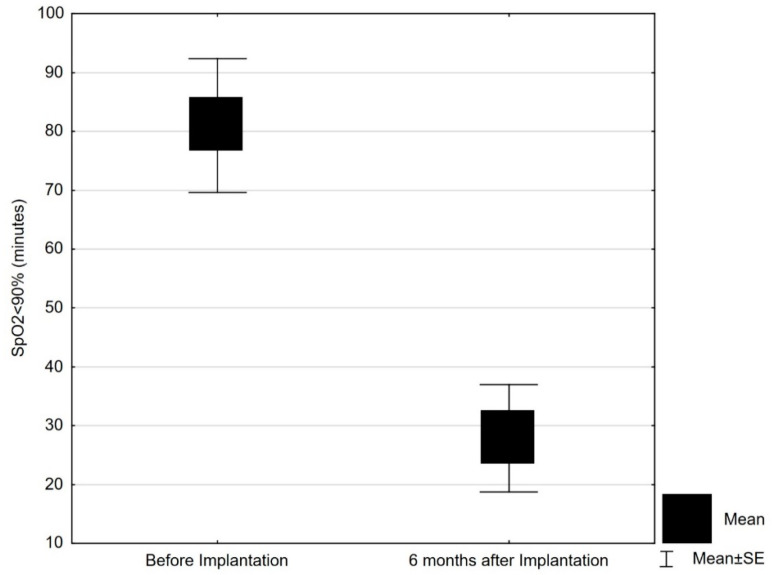
Hypoxemic burden (time with oxygen saturation below 90%) at baseline and after 6 months of phrenic nerve stimulation (mean with standard error). SpO_2_, oxygen saturation; SE, standard error.

Central respiratory events, both apneas and hypopneas, were well controlled by PNS. The number of total central apnea events decreased significantly from 109.5 ± 102.5 at baseline to 38.6 ± 53.5 six months after PNS implantation (*p* = 0.027) (Table 3). Initial Apnea-hypopnea index (AHI) was 38.1 ± 17.9/h and through phrenic nerve stimulation AHI showed 17.3 ± 9.4/h. Hypoxemic burden, determined based on the time with oxygen saturation <90% (T < 90) on PSG was 81 ± 55.7 min at baseline, and decreased to 27.8 ± 42.7 min during follow-up on PNS (*p* < 0.01) (Table 3, Figure 2).

### 3.3. Other Assessments

Left atrial diameter on echocardiography increased significantly during the study (*p* = 0.04) (Table 3), without any increase in atrial arrhythmias. Left ventricular ejection fraction and laboratory parameters including brain natriuretic peptide levels, C-reactive protein and kidney function (serum creatinine) remained unchanged throughout the study (Table 3).

### 3.4. Adverse Events

No serious adverse events occurred during the six-month study follow up in our study population.

## 4. Discussion

The two main endpoints in our study are related to mortality in patients with HF, while this trial did not investigate mortality per se, but two established surrogate markers in this field. Both physical capacity (determined using a standardized 6-min walk test) and hypoxemic burden (based on time with oxygen saturation of < 90% on PSG) showed significant improvement from baseline after implantation of a novel PNS device. Our findings also confirm previous data showing a reliable and persistent long-term reduction in AHI during treatment with PNS [11,13]. For the limited number of patients available for such a trial, we have not been able to show HF associated benefits in LVEF, BNP, or renal function, all these parameters remained unchanged. Despite documentation of left atrial gain, representing expected HF progression over time, our study provides data on mortality relevant endpoints.

Reducing mortality in patients with HF is an ongoing goal in clinical practice because these individuals remain at high risk of death despite significant advances in medical and device therapy [16]. Therefore, there is an unmet need for new approaches for treatment that have beneficial effects on mortality. In the largest trial to date in the field (*n* = 963), total AHI failed to predict mortality in HF patients with sleep apnea, but hypoxemic burden was a robust predictor of mortality [10]. Therefore, therapies able to reduce the hypoxic burden have the potential to reduce mortality in patients with HF [11]. This means that the reduction in hypoxemic burden seen during PNS therapy in our study is likely to be highly clinically relevant. So far, a PNS therapy trial on mortality has not been conducted yet.

A recent meta-analysis highlighted the high predictive value of physical capacity for HF prognosis [18]. Data from 10,368 patients were analyzed and a highly significant association between the six-minute walk distance and HF mortality was identified. Each 1-m increase in the six-minute walk distance was associated with a 1% decrease in the risk of all-cause mortality and cardiovascular events (hazard ratio (HR) 0.99, 95% confidence interval (CI) 0.99–1.00; *p* < 0.01). In addition, six-minute walk distance cut-off values were significantly associated with mortality (HR 2.04; 95% CI 1.48–2.83) [18]. These data suggest that the improvements in physical capacity documented during treatment with PNS in our study have the potential to have a beneficial contribution to mortality in patients with HF.

Our study confirmed the effectiveness of PNS for reducing CSA in patients with HF. However, the value of treating CSA in patients with HF and reduced ejection fraction (HFrEF) was questioned by the results of the Treatment of Predominant Central Sleep Apnoea by Adaptive Servo Ventilation in Patients With Heart Failure (SERVE-HF) study [19]. In that trial, patients with HFrEF randomized to treatment with adaptive servo-ventilation (ASV) were more likely to experience endpoint events than those in the control group. However, it is not clear whether these findings are generalizable to other approaches to treating CSA in patients with HF. The mechanism of action of PNS differs from that of ASV because PNS induces physiologic negative intrathoracic pressures, rather than the positive airway pressure provided by ASV [13,20].

Key strengths of our study are its novelty and the inclusion of clinically relevant endpoints for patients with HF. However, several limitations need to be taken into account when interpreting the findings. Firstly, the sample size is small (24 patients). This is due to the fact that PNS is a relatively new therapy that is only available at a few clinical centers, thus limiting the number of patients available for inclusion. In addition, the study lacked a control group, meaning that there was no control for potential confounding variables or the natural disease course in our population. Finally, the results are only applicable to the setting in which the study was conducted, and cannot be generalized to smaller centers with less expertise in PNS.

## 5. Conclusions

Our study is the first to describe the beneficial effects of PNS on clinical endpoints related to mortality in patients with HF and CSA. Although the findings are promising, the clinical benefits of PNS therapy in this patient population needs to be determined in a large, randomized controlled study with robust and objective clinical endpoints, including mortality.

## Figures and Tables

**Table 1:** Patient demographic and clinical characteristics at baseline.

Variable	Patients (*n* = 24)
Age, years	67.1 ± 11.2
Male, *n* (%)	22 (92)
Body mass index, kg/m^2^	34.6 ± 2.3
NYHA class II, *n* (%)	15 (62%)
NYHA class III, *n* (%)	9 (38%)
Sinus rhythm, *n* (%)	14 (56)
Ischemic etiology	15 (63)
LVEF, % (all patients)	42.4 ± 13.4
LVEF, % (20 HFrEF patients)	31.6 ± 11.8
LVEF, % (4 HFpEF patients)	54.1 ± 9.2
Left atrial diameter (mm)	51.4 ± 8.2
Pacemaker implanted, *n* (%)	1 (4)
ICD, *n* (%)	5 (21)
CRT, *n* (%)	4 (17)
Beta Blocker therapy, *n* (%)	22 (92)
ACE inhibitor therapy, *n* (%)	13 (54)
ARB therapy, *n* (%)	4 (17)
MRA therapy, *n* (%)	12 (50)

Values are mean ± standard deviation or number of patients (%). ACE, Angiotensin-converting-enzyme; ARB, Angiotensin II receptor blocker; CRT, Cardiac resynchronization therapy; HFrEF, heart failure with reduced ejection fraction; HFpEF, heart failure with preserved ejection fraction; ICD, implantable cardioverter-defibrillator; LVEF, left ventricular ejection fraction; MRA, Mineralocorticoid receptor antagonist; NYHA, New York Heart Association.

**Table 2:** Phrenic nerve stimulation interventional data.

	Interventional Data (*n* = 24)
Right pectoral implantation, *n* (%)	6 (25)
Implantation time, min	137.6 ± 50.4
Radiation, cGy*cm^2^	2279.5 ± 1553.2
Bipolar electrode, *n* (%)	7 (29)
Quadripolar electrode, *n* (%)	14 (58)
Right sided electrode, *n* (%)	4 (17)
Electrode sensing, mV	0.8 ± 0.4

Values are mean ± standard deviation or number of patients (%).

**Table 3:** Summary of study findings at baseline and 6-month follow-up.

Variables	Before Implantation	6-Month Follow-Up	*p*-Value
6-min walking distance, m	369.5 ± 163.5	410 ± 169.7	0.035
Time with SpO_2_ < 90%, min	81 ± 55.7	27.86 ± 42.7	<0.01
Time with SpO_2_ < 90%, %	19.5 ± 12.6	8.1 ± 9.8	<0.01
LVEF, %	42.4 ± 13.4	41.9 ± 14.7	0.89
Left atrial diameter, mm	51.4 ± 8.1	56.7 ± 7.8	0.04
BNP, pg/mL	630.4 ± 1682.8	835.4 ± 2045.3	0.74
CRP, mg/dL	0.5 ± 0.7	0.7 ± 0.9	0.43
Creatinine, mg/dL	1.2 ± 0.3	1.4 ± 0.7	0.23
Central respiratory events total	109.5 ± 102.5	38.6 ± 53.5	0.027
Total recording time, min	438 ± 61.1	448.6 ± 69.5	0.58
Central apnea index (/h)	18 ± 16.8	7.2 ± 10	0.02
Obstructive apnea index (/h)	2 ± 2.4	5.7 ± 9	0.2
Hypopnea index (/h)	14.5 ± 9.7	17.9 ± 15.8	0.39
Apnea-Hypopnea Index (/h)	38.1 ± 17.9	17.3 ± 9.4	0.01

Values are mean ± standard deviation. BNP, brain natriuretic peptide; CRP, C-reactive protein; LVEF, left ventricular ejection fraction; SpO_2_, oxygen saturation.

## Data Availability

All data is contained within this article.
